# Obestatin and growth hormone reveal the interaction of central obesity and other cardiometabolic risk factors of metabolic syndrome

**DOI:** 10.1038/s41598-020-62271-w

**Published:** 2020-03-26

**Authors:** Angus P. Yu, Felix N. Ugwu, Bjorn T. Tam, Paul H. Lee, Vicki Ma, Simon Pang, Angel S. Chow, Kenneth K. Cheng, Christopher W. Lai, Cesar S. Wong, Parco M. Siu

**Affiliations:** 1 Division of Kinesiology, School of Public Health, Li Ka Shing Faculty of Medicine, The University of Hong Kong, Pokfulam, Hong Kong, China; 2 Department of Health, Kinesiology and Applied Physiology, Concordia University, Montreal, QC Canada; 3 Department of Health Technology and Informatics, Faculty of Health and Social Sciences, The Hong Kong Polytechnic University, Hung Hom, Kowloon, Hong Kong, China; 4 School of Nursing, Faculty of Health and Social Sciences, The Hong Kong Polytechnic University, Hung Hom, Kowloon, Hong Kong, China; 5 Singapore Institute of Technology, Singapore, Singapore

**Keywords:** Cytokines, Metabolic syndrome

## Abstract

Metabolic syndrome (MetS) is a multi-factorial disorder including central obesity (CO), insulin resistance, hyperglycemia, dyslipidemia and hypertension which increases the risk of diabetes mellitus and cardiovascular diseases. CO is considered as an essential component of MetS according to International Diabetes Federation (IDF), which may further modulate distinct signalling pathways compared with the other four MetS risk factors. Given that ghrelin signalling and the growth hormone/insulin-like growth factor-1 (GH/IGF-1) axis regulates energy balance and metabolic homeostasis, this study examined the changes in various ghrelin products and circulating hormones in response to the interaction between CO and other MetS components including blood pressure, fasting blood glucose, triglycerides, and high-density lipoprotein cholesterol in 133 Hong Kong Chinese adults. Circulating obestatin and GH were increased and reduced, respectively, by either CO or the other 4-risk factor cluster. These changes were further augmented by the presence of all MetS risk factors. However, changes of ghrelin levels were not mediated by CO but the other MetS risk factors. Our findings suggest that CO does not predict all the dysregulation of signalling pathways in individuals with MetS. Although CO and other MetS may share common signalling targets (i.e., obestatin and GH), CO does not contribute to the perturbation of ghrelin signalling.

## Introduction

Individuals with metabolic syndrome (MetS) exhibiting concomitant aberrant cardiometabolic homeostasis are prone to the development of chronic diseases such as cardiovascular diseases, diabetes mellitus and stroke. MetS is a collection of metabolic risk factors including central obesity (CO), high blood pressure, hyperglycemia, and dyslipidemia^1^. To date, there is no consensus in the diagnosis of MetS. According to the National Cholesterol Education Program-Third Adult Treatment Panel III (NCEP-ATP III), a MetS-positive individual should manifest 3 risk factors in any combination whereas the International Diabetes Federation (IDF) recommended the inclusion of CO in conjunction with any other two risk factors among elevated blood pressure, elevated triglycerides, elevated fasting blood glucose and reduced high-density lipoprotein-cholesterol (HDL-C)^2^. Although the contribution of respective risk factors to the prevalence of MetS has been studied, it remains unknown whether CO, as proposed by IDF, is a critical key risk factor preceding to the development MetS. CO is indicative of accumulated intra-abdominal adiposity and associated with the presentation of other MetS cardiovascular risk factors^3^. Indeed, individuals without confirmed MetS but presenting CO are more likely to exhibit increased risk of cardiovascular diseases^4^. Given that each risk factor may alter specific molecular pathways that lead to the pathogenesis of cardiometabolic disorders, it is speculated that CO may contribute to the development of cardiovascular diseases, diabetes and cancers through certain mechanisms that are yet to be identified. More importantly, whether CO exhibits synergism with other risk factors or a distinct, predominating role in the development of MetS have not been addressed.

Ghrelin signaling and the growth hormone/insulin-like growth factor-1 (GH/IGF-1) axes are essential to metabolic health through the regulation of energy balance and metabolic homeostasis. A computational study that was designed to mimic human-like metabolic system has deciphered the presence of positive energy balance (i.e., more energy intake than expenditure) during the development of MetS^5^. Persistent exhibition of positive energy balance is associated with the development of MetS, cardiovascular diseases and type 2 diabetes^6,7^. The ghrelin gene products, namely acylated ghrelin (AG), unacylated ghrelin (UnAG) and obestatin control energy intake through the regulation of appetite^8^. GH, IGF-1 and nesfatin-1 of the GH/IGF-1 axis are implicated in carbohydrate and lipid metabolism^9^. It is noteworthy that these signalling components are associated with the regulation of blood pressure, glucose homeostasis and lipid profile^10–16^. Given that metabolic homeostasis is regulated intricately by ghrelin signaling and the GH/IGH-1 axis, we hypothesized that CO, which is an essential MetS risk factor as defined by IDF, may contribute to the disruption of ghrelin signaling and the GH/IGH-1 axis in addition to the other four MetS risk factors. This study aimed to dissect how the alteration of ghrelin signaling pathway and GH/IGH-1 axis by CO is different from those induced by other MetS risk factors in order to understand the underlying mechanisms through which CO facilitates the development of cardiovascular diseases and metabolic disorders.

## Methods

### Subjects and MetS parameters

Based on specific criteria for MetS parameters defined by NCEP-ATP III, serum and MetS data of 133 Hong Kong Chinese adults of both sexes with age ranged 24 to 86 years were retrieved from a total of 1492 archived data of participants screened between November 2010 and August 2013^1^. Notably, MetS is a collection of cardiovascular risk factors, suggesting individuals with MetS may manifest different combinations of MetS risk factors. The heterogeneity of the MetS risk factors thus, imposes challenges in analyses and interpretation of the signalling peptides studied. In this study, only the samples from individuals who have: 1) no MetS risk factor (NRFNO, n = 53), 2) central obesity without any other MetS risk factors (NRFO, n = 33), 3) concomitant exhibition of four MetS risk factors (RFNO, n = 10) and 4) all MetS risk factors (RFO, n = 37) as defined by NCEP-ATP III [i.e., (1) central obesity is indicated by waist circumference (WC) exceeding 90 cm or 80 cm for Asian males and females, respectively, (2) high blood pressure is defined by >130 mmHg systolic blood pressure (SBP) or >85 mmHg diastolic blood pressure (DBP), (3) fasting blood glucose (FBG) is defined with >5.5 mmol/L, (4) elevated plasma triglycerides (TG) ≥1.7 mmol/L, and (5) low level of high-density lipoprotein cholesterol (HDL-C) less than 1.03 mmol/L or 1.29 mmol/L for males or females, respectively^17^] were selected in order to investigate the effect of CO alone and its interaction with the other four MetS risk factors on ghrelin signaling and the GH/IGH-1axis. Hence, a 2 × 2 factorial design (central obesity X the cluster of the other four MetS factors) was adopted to evaluate the interaction of central obesity with the cluster of the other four MetS parameters namely elevated blood pressure, elevated triglycerides, elevated fasting blood glucose and reduced HDL-C. All subjects were screened and individuals with dementia or mental disorders, severe or acute cardiovascular diseases, post-stroke, neuromusculoskeletal illness, acute medical illness, symptomatic heart or lung diseases, severe rheumatoid arthritis, osteoarthritis or pulmonary illness and participants who were immobile, smoker or under treatment for metabolic abnormalities were excluded. Data were analyzed by classifying the participants into: (A) absence of the cluster of four MetS risk factors including elevated blood pressure, elevated triglycerides, elevated fasting blood glucose, reduced HDL-C (i.e., NRFNO and NRFO) vs. presence of the cluster of four MetS risk factors (i.e. RFNO and RFO); (B) non-central obese (i.e., NRFNO and RFNO) vs. central obese (i.e., NRFO and RFO); (C) absence of any MetS risk factors (NRFNO) vs. central obesity but no other MetS risk factors (NRFO) vs. presence of the cluster of the four MetS risk factors but without central obesity (RFNO) vs. presence of both the cluster of the four MetS risk factors and central obesity (RFO). Blood pressure (systolic and diastolic) was determined by an electronic blood pressure monitor (Accutorr Plus, Datascope). Waist circumference was measured by an inelastic measuring tape by trained personnel. Fasting venous blood samples were obtained after at least 10 hours fast by certified phlebotomists. Serum blood samples were sent to an accredited medical laboratory for the measurements of fasting blood glucose, triglycerides and HDL-C by commercial test kits using an automatic clinical chemistry analyzer (Architect CI8200, Abbott Diagnostics). All samples were aliquoted and stored at −80 °C until needed for analysis. Human research ethics approval was obtained from the human subject ethics subcommittee of the Hong Kong Polytechnic University (ethics approval number: HSEARS20150203002) and written informed consent was obtained from the subjects prior to commencement of the experiment. All methods were performed in accordance with the relevant guidelines and regulations.

### Peptide measurement

#### Enzyme-linked immunosorbent assay (ELISA)

Sera from the obtained venous blood samples were used to measure UnAG, AG, obestatin, nesfatin-1, GH and IGF-1 by commercially available ELISA kits. UnAG and AG kits were purchased from BioVendor – Laboratiorni medicina a.s., Karasek, Czech Republic (RA194063400R and RA194062400R, respectively). The detection limit of the UnAG and AG kits were 0.2 pg/ml and.0.3 pg/ml respectively. Obestatin and nesfatin-1 kits were purchased from Biomatik Corporation, Canada (EKU06381 and EKU06180, respectively). The detection limit of the obestatin and nesfatin-1 kits were 29 pg/ml and 234.2 pg/ml. GH and IGF-1 kits were obtained from Abcam, Cambri (ab190811 and ab100545, respectively). The detection limit of the GH and IGF-1 kits were 1.6 pg/ml and <0.2 ng/ml. All protocols were in accordance with manufacturers´ recommendations.

### Statistical analysis

Data are expressed as mean ± standard deviation. Chi-square test was employed to detect the difference of gender ratio among groups. Normality of the data was tested by Kolmogorov-Smimov test and reveled that our data was not normally distributed. The main effect of CO, the main effect of the cluster of the other four MetS risk factors and the interaction of CO with the cluster of the other four MetS risk factors on UnAG, AG, total ghrelin, obestatin, nesfatin-1, GH and IGF-1 and the ratio of obestatin/ghrelin were analyzed by generalized estimating equations (GEE). GEE was adopted as it has less stringent requirement on normality assumption. The differences between two groups were detected by Mann-Whitney U Test whereas statistical differences among the four groups were determined by Kruskal-Wallis H Test, followed by Dunn-Bonferroni post hoc tests. Spearman’s correlation analysis was performed to examine the correlations between the peptide concentrations and the MetS parameters. Multivariate linear regression was conducted using MetS risk factors as independent variable and the concentration of circulating concentration as dependent variable to see how MetS risk factors alter the mediators of ghrelin signalling and GH/IGF-1 axis. Statistical significance was accepted at p < 0.05. All statistical procedures were conducted using the Statistical Package for the Social Sciences (SPSS) version 26 for Windows.

## Results

### Characteristics of participants of selected samples

The characteristics of studied participants were summarised in Table 1. As expected, systolic and diastolic blood pressures, fasting blooding glucose and triglycerides in RFNO and RFO were significantly higher than NRFNO and NRFO (all p < 0.001) whereas HDL-C was significantly lower in RFNO and RFO compared with NRFNO and NRFO (all p < 0.001). Likewise, waist circumferences in NRFO and RFO was significantly larger than NRFNO and RFNO (all p < 0.005). The waist circumference of RFO was remarkably larger than NRFO (p < 0.001) Notably, the age of RFO was significantly larger than NRFNO (p = 0.033). The gender ratio was not significantly different among groups (p = 0.396).

**Table 1:** Characteristics of participants.

	NRFNO	NRFO	RFNO	RFO
n	53	33	10	37
Female (%)	81	79	60	70
Age (years)	61.13 ± 5.35	57.72 ± 9.73	64.10 ± 4.93	63.54 ± 12.06
SBP (mmHg)	117.58 ± 7.33	120.09 ± 8.67	170.50 ± 10.6	157.95 ± 20.68
DP (mmHg)	70.26 ± 5.69	71.00 ± 6.09	82.00 ± 8.71	85.89 ± 10.08
WC (cm)	74.45 ± 5.61	86.60 ± 4.50	80.10 ± 4.46	92.73 ± 9.75
TG (mmol/L)	1.05 ± 0.30	1.06 ± 0.28	2.27 ± 0.52	2.55 ± 0.92
FBG (mmol/L)	4.96 ± 0.31	5.08 ± 0.29	6.56 ± 0.77	6.87 ± 1.36
HDL-C (mmol/L)	1.69 ± 0.35	1.65 ± 0.33	1.04 ± 0.16	0.99 ± 0.15

NRFNO, group of participants with no cardiometabolic risk factors; NRFO: group of participants with central obesity but no other cardiometabolic risk factors; RFNO: group of participants with no central obesity but all other four cardiometabolic risk factors: RFO: group of participants with all cardiometabolic risk factors; SBP, systolic blood pressure; DBP, diastolic blood pressure; WC, waist circumference; TG, triglycerides; FBG, fasting blood glucose; HDL-C, high-density lipoprotein cholesterol.

### Obestatin but not ghrelin revealed the interaction of CO with the cluster of the other four MetS risk factors

**Figure 1:**
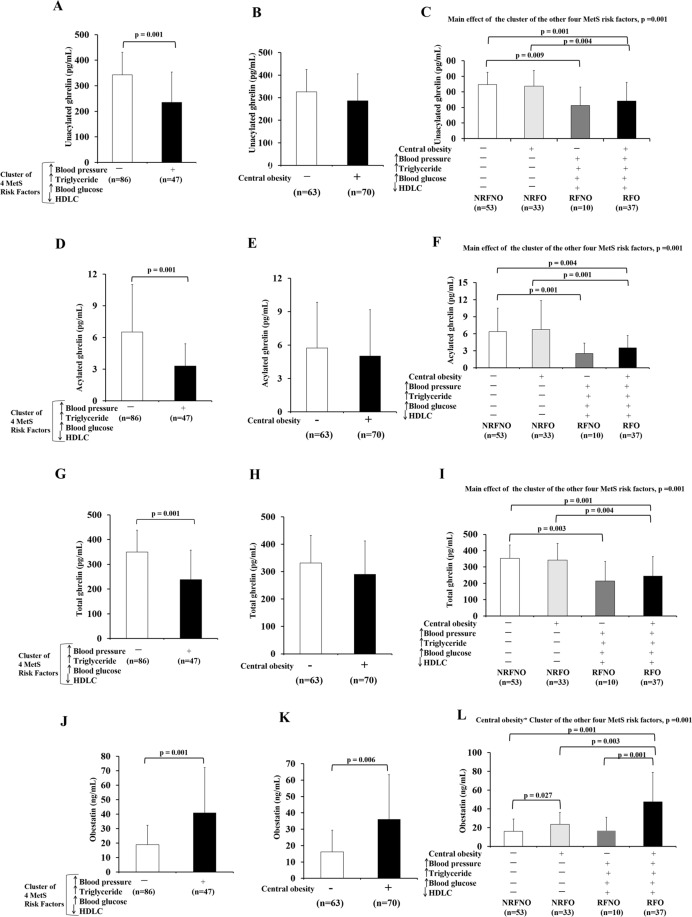
Levels of ghrelin gene products in response to CO or the cluster of the other four MetS risk factors alone as well as CO in conjunction with the cluster of the other four MetS risk factors. Unacylated ghrelin, acylated ghrelin and total ghrelin levels of subjects with/without the cluster of four MetS risk factors regardless of the status of CO (RFNO and RFO vs NRFNO and NRFO) (**A,D,G**). Unacylated ghrelin, acylated ghrelin and total ghrelin levels of subjects with/without CO regardless of the status of the cluster of four MetS risk factors (NRFNO and RFNO vs NRFO and RFO) (**B,E,H**). Comparison of unacylated ghrelin, acylated ghrelin and total ghrelin levels among subjects with no MetS risk factors, central obese only, the cluster of the other MetS risk factors other than central obese, and all MetS risk factors (**C,F,I**). Obestatin levels of subjects with/without the cluster of four MetS risk factors regardless of the status of CO (RFNO and RFO vs NRFNO and NRFO) (**J**). Obestatin levels of subjects with/without CO regardless of the status of the cluster of four MetS risk factors (NRFNO and RFNO vs NRFO and RFO) (**K**). Comparison of obestatin among subjects with no MetS risk factors (NRFNO), central obese only (NRFO), the cluster of the other MetS risk factors other than central obese (RFNO), and all MetS risk factors (RFO) (**L**).

The level of UnAG was 32% (mean difference = 108 pg/mL) significantly reduced in subjects with the cluster of four MetS risk factors compared to subjects without the cluster of four MetS risk factors (p = 0.001) (Fig. 1A). The main effect of the cluster of the other four MetS risk factors on UnAG (p = 0.001) was statistically significant (Fig. 1C). Significant differences in UnAG were observed between NRFNO and RFNO (p = 0.009), NRFNO and RFO (p = 0.001) and between NRFO and RFO groups (p = 0.004) (Fig. 1C).

AG was 49% (mean difference = 3.2 pg/mL) significantly lower in subjects with the cluster of four MetS risk factors compared to subjects without the cluster of four MetS risk factors (p = 0.001) (Fig. 1D). The main effect of the cluster of the other four MetS risk factors on AG was statistically significant (p = 0.001) (Fig. 1F). Significant differences in AG were found between NRFNO and RFNO (p = 0.001), NRFNO and RFO (p = 0.004) and between NRFO and RFO groups (p = 0.001) (Fig. 1F).

Total ghrelin was 32% (mean difference = 111.3 pg/mL) significantly reduced in subjects with the cluster of four MetS risk factors compared to subjects without the cluster of four MetS risk factors (p = 0.001) (Fig. 1G). The main effect of the cluster of the other four MetS risk factors on total ghrelin was statistically significant (p = 0.001) (Fig. 1I). Significant differences in total ghrelin were observed between NRFNO and RFNO (p = 0.003), NRFNO and RFO (p = 0.001) and between NRFO and RFO groups (p = 0.004) (Fig. 1I).

In contrast to UnAG, AG and total ghrelin, obestatin was 116% (mean difference: 22 ng/mL) significantly higher in subjects with the cluster of four MetS risk factors compared to subjects without the cluster of four MetS risk factors (p = 0.001) (Fig. 1J). Also, obestatin was 123% (mean difference: 20 ng/mL) significantly higher in central obese subjects when compared with non-central obese subjects (p = 0.001) (Fig. 1K). CO and the cluster of the other four MetS risk factors had interaction effect on obestatin (p = 0.001) (Fig. 1L). Significant differences were observed between NRFNO and NRFO (p = 0.027), NRFNO and RFO (p = 0.001), NRFO and RFO (p = 0.003) and between RFNO and RFO groups (p = 0.001) (Fig. 1L). The differences in obestatin levels among groups were valided by immunoblotting (Supplementary Fig. 1A–C).

### Ratios of ghrelin gene products reflect the interaction of CO with the cluster of the other four MetS risk factors

**Figure 2:**
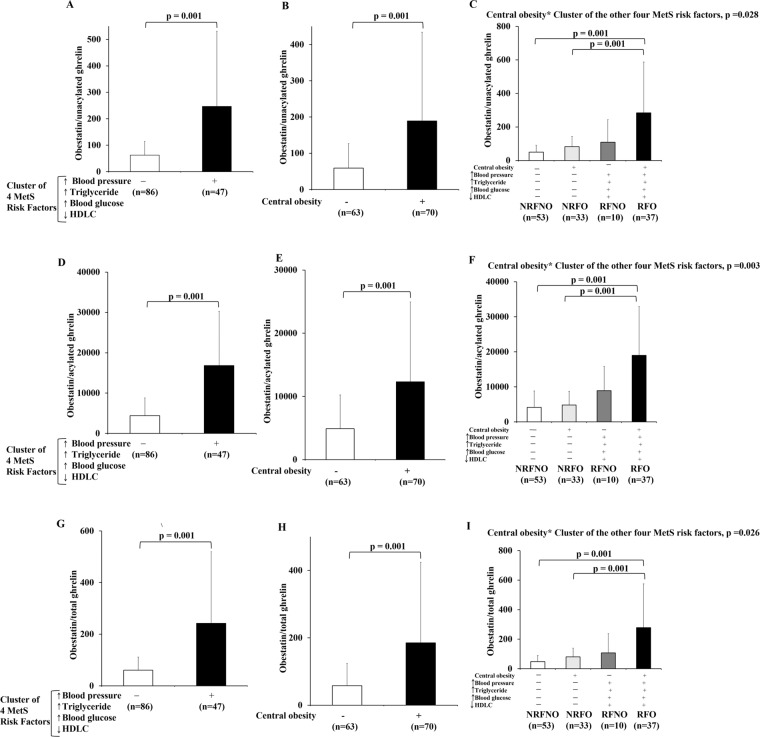
Ratios of ghrelin gene products in response to CO or the cluster of the other four MetS risk factors alone as well as CO in conjunction with the cluster of the other four MetS risk factors. The ratios of obestatin/unacylated ghrelin, obestatin/acylated ghrelin and obestatin/total ghrelin of subjects with/without the cluster of four MetS risk factors regardless of the status of CO (RFNO and RFO vs NRFNO and NRFO) (**A,D,G**). The ratios of obestatin/unacylated ghrelin, obestatin/acylated ghrelin and obestatin/total ghrelin of subjects with/without CO regardless of the status of the cluster of four MetS risk factors (NRFNO and RFNO vs NRFO and RFO) (**B,E,H**). Comparison of ratios of obestatin/unacylated ghrelin, obestatin/acylated ghrelin and obestatin/total ghrelin among subjects with no MetS risk factors (NRFNO), central obese only (NRFO), the cluster of the other MetS risk factors other than central obese (RFNO), and all MetS risk factors (RFO) (**C,F,I**).

In the current study, the ratio of obestatin/UnAG was significantly higher in subjects with the cluster of four MetS risk factors compared to subjects without the cluster of four MetS risk factors by 4-fold (p = 0.001) (Fig. 2A). Similarly, the ratio of obestatin/UnAG was significantly higher in central obese subjects when compared to non-central obese subjects by 3.2-fold (p = 0.001) (Fig. 2B). CO and the cluster of the other four MetS risk factors had interaction effect (p = 0.028) on the ratio of obestatin/UnAG (Fig. 2C). Post-hoc tests revealed significant differences in the ratio of obestatin/UnAG between NRFNO and RFO (p = 0.001) and between NRFO and RFO groups (p = 0.001) (Fig. 2C).

The ratio of obestatin/AG was significantly higher in subjects with the cluster of four MetS risk factors compared to subjects without the cluster of four MetS risk factors by 3.8-fold (p = 0.001) (Fig. 2D). Similarly, the ratio of obestatin/AG was significantly higher in central obese subjects when compared to non-central obese subjects by 2.5-fold (p = 0.001) (Fig. 2E). CO and the cluster of the other four MetS risk factors had interaction effect (p = 0.003) on the ratio of obestatin/AG (Fig. 2F). Post-hoc tests revealed significant differences in the ratio of obestatin/AG between NRFNO and RFO (p = 0.001) and between NRFO and RFO groups (p = 0.001) (Fig. 2F).

The ratio of obestatin/total ghrelin was significantly higher in subjects with the cluster of four MetS risk factors compared to subjects without the cluster of four MetS risk factors by 4-fold (p = 0.001) (Fig. 2G). Similarly, the ratio of obestatin/total ghrelin was significantly higher in central obese subjects when compared to non-central obese subjects by 3.2-fold (p = 0.001) (Fig. 2H). CO and the cluster of the other four MetS risk factors had interaction effect (p = 0.026) on the ratio of obestatin/total ghrelin (Fig. 2I). Post-hoc tests revealed significant differences in the ratio of obestatin/total ghrelin between NRFNO and RFO (p = 0.001) and between NRFO and RFO groups (p = 0.001) (Fig. 2I).

### GH but not Nesfatin-1 or IGF-1 revealed the interaction of CO with the cluster of the other four MetS risk factors

**Figure 3:**
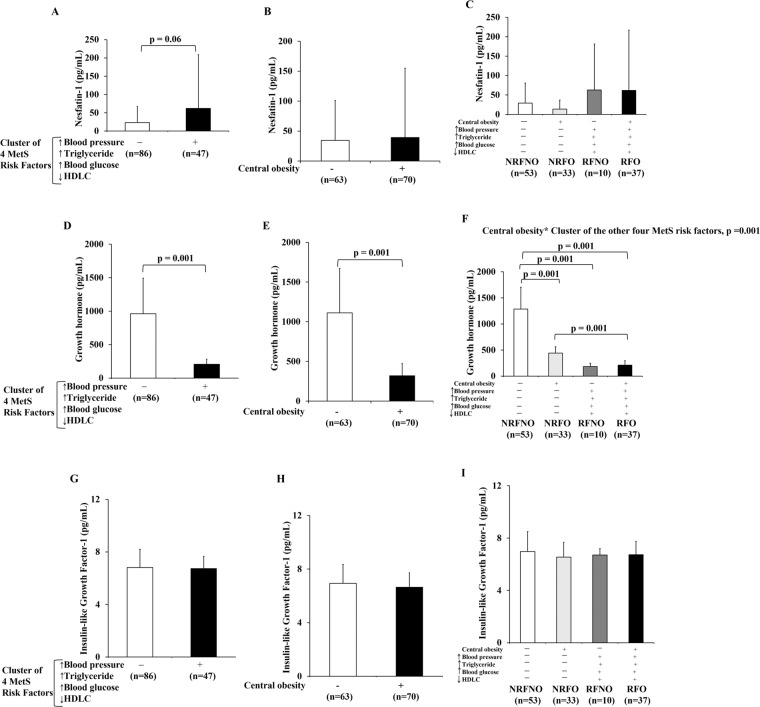
Levels of nesfatin-1, GH and IGF-1 in response to CO or the cluster of the other four MetS risk factors alone as well as CO in conjunction with the cluster of the other four MetS risk factors. Nesfatin-1 level of subjects with/without the cluster of four MetS risk factors regardless of the status of CO (RFNO and RFO vs NRFNO and NRFO) (**A**). Nesfatin-1 level of subjects with/without CO regardless of the status of the cluster of four MetS risk factors (NRFNO and RFNO vs NRFO and RFO) (**B**). Comparison of Nesfatin-1 level among subjects with no MetS risk factors (NRFNO), central obese only (NRFO), the cluster of the other MetS risk factors other than central obese (RFNO), and all MetS risk factors (RFO) (**C**). Growth hormone level of subjects with/without the cluster of four MetS risk factors regardless of the status of CO (RFNO and RFO vs NRFNO and NRFO) (**D**). Growth hormone level of subjects with/without CO regardless of the status of the cluster of four MetS risk factors (NRFNO and RFNO vs NRFO and RFO) (**E**). Comparison of growth hormone level among subjects with no MetS risk factors (NRFNO), central obese only (NRFO), the cluster of the other MetS risk factors other than central obese (RFNO), and all MetS risk factors (RFO) (**F**). Insulin-like growth factor-1 level of subjects with/without the cluster of four MetS risk factors regardless of the status of CO (RFNO and RFO vs NRFNO and NRFO) (**G**). Insulin-like growth factor-1 level of subjects with/without CO regardless of the status of the cluster of four MetS risk factors (NRFNO and RFNO vs NRFO and RFO) (**H**). Comparison of Insulin-like growth factor-1 level among subjects with no MetS risk factors (NRFNO), central obese only (NRFO), the cluster of the other MetS risk factors other than central obese (RFNO), and all MetS risk factors (RFO) (**I**).

Nesfatin-1 was not significantly higher in subjects with the cluster of four MetS risk factors compared to subjects without the cluster of four MetS risk factors (Fig. 3A). No statistical difference in Nesfatin-1 was observed in participants with CO and those without CO (Fig. 3B). Neither interactions nor main effects of CO with the cluster of the other four MetS risk factors was not exhibited by nesfatin-1 (Fig. 3C).

There was neither significant difference in IGF-1 level between subjects with the cluster of four MetS risk factors and subjects without the cluster of four MetS risk factors (Fig. 3G) nor between central obese and non-central obese subjects (Fig. 3H). There was no interaction of CO with the cluster of the other four MetS risk factors on IGF-1 (Fig. 3I). Also, there was no main effect of CO or the cluster of the other four MetS risk factors on IGF-1 (Fig. 3I).

GH was 79% significantly lower in subjects with the cluster of four MetS risk factors in comparison to subjects without the cluster of four MetS risk factors(mean difference = 756.9 pg/mL, p = 0.001) (Fig. 3D). GH was 71% (mean difference = 792.3 pg/mL, p = 0.001) significantly lower in central obese individuals compared to non-central obese subjects (Fig. 3E). CO and the cluster of the other four MetS risk factors had interaction effect on GH (p = 0.001) (Fig. 3F). Post-hoc tests showed significant difference in GH between NRFNO and NRFO (p = 0.001), NRFNO and RFNO (p = 0.001), NRFNO and RFO (p = 0.001) and between NRFO and RFO groups (p = 0.001) (Fig. 3F). The differences in GH levels among groups were valided by immunoblotting (Supplementary Fig. 1D–F).

### Relationship of biomarker with cardiometabolic risk factors

The associations of UnAG, AG, total ghrelin, obestatin, nesfatin-1, GH and IGF-1 with the individual cardiometabolic risk factors were presented as correlation coefficients in Table 2. UAG, AG, total ghrelin, GH were positively associated with SBP, DBP, WC, FBG and TG but negatively associated with HDL-C (all p < 0.05). Obestatin were negatively associated with SBP, DBP, WC, FBG and TG but positively associated with HDL-C (all p < 0.05). Nesfatin-1 was positively associated with triglycerides (p = 0.008). There was no correlation between IGF-1 and all cardiometabolic risk factors.

**Table 2:** Correlation between ghrelin gene products, nesfatin-1, GH, IGF-1 and cardiometabolic risk factors.

	UnAG	AG	Total Ghrelin	Obestatin	Nesfatin-1	GH	IGH-1
SBP	ρ = −0.426 p < 0.001	ρ = −0.476 p < 0.001	ρ = −0.446 p < 0.001	ρ = 0.370 p < 0.001	ρ = 0.095 p = 0.278	ρ = −0.693 p < 0.001	ρ = 0.062 p = 0.475
DBP	ρ = −0.214 p = 0.013	ρ = −0.304 p < 0.001	ρ = −0.220 p = 0.011	ρ = 0.220 p = 0.011	ρ = 0.116 p = 0.183	ρ = −0.610 p < 0.001	ρ = −0.001 p = 0.987
WC	ρ = −0.331 p < 0.001	ρ = −0.180 p = 0.038	ρ = −0.339 p < 0.001	ρ = 0.440 p < 0.001	ρ = 0.068 p = 0.438	ρ = −0.646 p < 0.001	ρ = −0.119 p = 0.174
TG	ρ = −0.383 p < 0.001	ρ = −0.344 p < 0.001	ρ = −0.388 p < 0.001	ρ = 0.384 p < 0.001	ρ = 0.231 p = 0.008	ρ = −0.702 p < 0.001	ρ = −0.044 p = 0.615
FBG	ρ = −0.419 p < 0.001	ρ = −0.410 p < 0.001	ρ = −0.377 p < 0.001	ρ = 0.373 p < 0.001	ρ = 0.110 p = 0.207	ρ = −0.693 p < 0.001	ρ = −0.037 p = 0.676
HDL−C	ρ = 0.367 p < 0.001	ρ = 0.329 p < 0.001	ρ = 0.422 p < 0.001	ρ = −0.323 p < 0.001	ρ = −0.151 p = 0.082	ρ = 0.668 p < 0.001	ρ = 0.092 p = 0.294

UnAG, AG, total ghrelin and GH were positively correlated with HDLC only but negatively correlated with the remaining cardiometabolic risk factors (SBP, DBP, WC, TG and FBG). Obestatin correlated negatively with HDLC but correlated positively with all other MetS risk factors. Nesfatin-1 correlated positively with TG alone whereas, IGF-1 showed no correlation with all MetS risk factors. UnAG, unacylated ghrelin; AG, acylated ghrelin; SBP, systolic blood pressure; DBP, diastolic blood pressure; WC, waist circumference; TG, triglycerides; FBG, fasting blood glucose; HDL-C, high-density lipoprotein cholesterol; Spearman’s correlation was employed for the correlation analysis. Significance level was set at p < 0.05.

The results of multivariate linear regression analysis, presented as unstandardized coefficients, were listed in Table 3. Age was a predictive variable of UnAG (p = 0.009) and total ghrelin (p = 0.009). Gender was a predictive variable of obestatin (p < 0.001). SBP was a predictive variable of UnAG (p = 0.004), AG (p = 0.027), total ghrelin (p = 0.003) and GH (p = 0.020). WC was a predictive variables of obestatin (p < 0.001), GH (p < 0.001) and nesfatin-1 (p = 0.002).

**Table 3:** Summary of multivariate linear regression of ghrelin gene products, nesfatin-1, GH and IGF-1 with age, gender and cardiometabolic risk factors.

r^2^	UnAG		AG		Total Ghrelin		Obestatin		Nesfatin-1		GH		IGF-1	
	0.362		0.180		0.369		0.440		0.198		0.530		0.019	
	B	P value	B	P value	B	P value	B	P value	B	P value	B	P value	B	P value
Gender	−10.36 ± 21.40	0.629	−1.48 ± 0.90	0.101	−11.84 ± 21.58	0.584	−25067.01 ± 4267.08	<0.001	−2.44 ± 20.44	0.905	158.08 ± 92.29	0.089	−0.11 ± 0.30	0.637
Age	−2.88 ± 1.09	0.009	−0.04 ± 0.05	0.339	−2.93 ± 1.10	0.009	42.20 ± 217.48	0.846	−1.99 ± 1.04	0.058	6.61 ± 4.70	0.162	−0.00 ± 0.02	0.823
SBP	−1.68 ± 0.57	0.004	−0.05 ± 0.02	0.027	−1.73 ± 0.57	0.003	190.70 ± 113.01	0.094	0.06 ± 0.54	0.919	−5.78 ± 2.44	0.020	0.01 ± 0.01	0.433
DBP	0.25 ± 1.11	0.825	−0.03 ± 0.05	0.530	0.22 ± 1.11	0.846	−153.79 ± 221.83	0.489	−0.71 ± 1.06	0.507	−1.64 ± 4.80	0.733	0.00 ± 0.02	0.827
WC	−1.27 ± 0.99	0.200	0.02 ± 0.04	0.646	−1.25 ± 1.00	0.210	1000.41 ± 196.81	<0.001	3.04 ± 0.94	0.002	−21.37 ± 4.26	<0.001	−0.00 ± 0.01	0.753
TG	−5.30 ± 13.83	0.702	−0.01 ± 0.58	0.985	−5.29 ± 13.95	0.705	3452.90 ± 2758.91	0.201	19.12 ± 13.22	0.150	−73.22 ± 59.67	0.222	−0.06 ± 0.19	0.756
FBG	5.92 ± 9.41	0.530	−0.43 ± 0.39	0.282	5.50 ± 9.49	0.563	78.22 ± 1875.85	0.067	14.58 ± 8.99	0.107	−23.63 ± 40.57	0.561	−0.08 ± 0.13	0.522
HDL−C	30.97 ± 29.40	0.295	−0.94 ± 1.23	0.447	30.04 ± 29.65	0.313	−3734.07 ± 5864.02	0.525	45.02 ± 28.09	0.112	169.66 ± 126.82	0.183	0.19 ± 0.41	0.649

UnAG, unacylated ghrelin; AG, acylated ghrelin; SBP, systolic blood pressure; DBP, diastolic blood pressure; WC, waist circumference; TG, triglycerides; FBG, fasting blood glucose; HDL-C, high-density lipoprotein cholesterol; B: unstandardized coefficient. Data was analyzed by multivariate linear regression. Significance level was set at p < 0.05.

## Discussion

Understanding the interactions among the MetS risk factors and the signalling pathways therein, may lead to conceptual advances in the etiology of MetS and ultimately the design of preventive strategies. The NCEP-ATP III has included CO as a diagnostic criterion of MetS^18^. In 2005, IDF has proposed that CO, measured as waist circumference, must be presented in any given confirmed cases of MetS^2^. The present study attempted to reveal whether the interaction between CO and the cluster of the other four MetS risk factors namely raised blood pressure, raised triglycerides, raised fasting blood glucose and reduced HDL cholesterol could alter the circulatory peptide profile related to ghrelin signalling and GH/IGF-1 axis including UnAG, AG, obestatin, nesfatin-1, GH and IGF-1. Our results have illustrated that the interaction of CO and the other four MetS risk factors altered the circulating levels of obestatin, obestatin/ghrelin ratios and GH whereas AG, UnAG and total ghrelin was only altered by the cluster of the other four MetS risk factors.

Ghrelin, a peripheral hormone that regulates orexigenic response and energy balance, has always been a subject of intense research in energy-related disorders. Due to the fact that the 2 forms of ghrelin may act in an identical or antagonistic manner, it is necessary to study and distinguish the physiological significance resulted from each member^19,20^. Ghrelin is known to regulate blood pressure via multiple signalling mechanisms^10^. Associations between ghrelin and insulin resistance and the level of HDL-C suggest that ghrelin is also implicated in the regulation of blood glucose and lipid^21^. Recent reports have proposed a correlation between chronic low-grade inflammation and disruption of energy homeostasis^22^. Moreover, the ghrelin signalling pathway may represent novel therapeutic opportunities for obesity-associated inflammatory diseases including diabetes and cancers due to the anti-inflammatory properties of various ghrelin gene products^23,24^. Our GEE analysis has shown that the circulating levels of AG, UnAG and total ghrelin were consistently diminished in individuals presenting the cluster of four MetS risk factors regardless of concomitant CO. Moreover, our multivariate linear regression analyses have demonstrated that CO, as determined by waist circumference, was not always appropriate to predict the changes in the ghrelin signalling molecules examined. All these data suggests that CO alone does not predict all the dysregulation of signalling pathways related to MetS, and the pathogenic mechanisms of cardiovascular diseases and metabolic perturbation in response to CO and the other four MetS risk factors may be dissimilar. While It has been demonstrated that CO alone is related to increased incidence of cardiovascular diseases^4^ and type 2 diabetes^25^, the absence of alterations in AG, UnAG and total ghrelin in centrally obese participants suggests that the effects of CO might be mediated by a ghrelin-independent, yet to be identified mechanism. Other obesity-related signalling pathways, such as the nitric oxide signalling, has been implicated in the development of cardiovascular diseases and type 2 diabetes^26,27^. Further research is warranted to identify the signalling pathways that link CO with the development of cardiovascular diseases and type 2 diabetes mellitus.

Although obestatin was initially identified as an appetite suppressant, the role of this ghrelin gene product in metabolism remains a subject of ongoing debate^28,29^. However, it is believed that obestatin is implicated in the regulation of metabolic homeostasis and cardiovascular function. Previous studies have demonstrated that obestatin increased beta cell mass and improved lipid metabolism; which may account for the inverse correlation between its circulating level with fasting blood glucose^11^. Our data have shown that individuals with CO manifested elevated circulating obestatin and obestatin/ghrelin ratio regardless of concurrent presentation of the four-MetS risk factor cluster. Likewise, the level of obestatin and obestatin/ghrelin ratio were higher in individuals presenting the four-MetS risk factor cluster and this was not affected by CO. Compared with the individuals without any MetS risk factors, CO alone but not the other 4 risk factors elevated the level of obestatin. Presentation of the other 4-risk factor cluster only elevated the obestatin level in individuals with CO. Given that the ratio of obestatin/ghrelin is also an indicator of energy-related metabolic disorders^30,31^, we have also included the corresponding analyses in our study. We observed that CO or the 4-risk factor cluster alone was not sufficient to increase the obestatin/ghrelin ratio but this ratio was elevated in individuals manifesting all the MetS risk factors studied. Our data thus indicate a synergistic effect of CO and the other 4 risk factors on the elevation of circulating obestatin through mechanisms remain to be identified.

GH is an important regulatory factor in metabolic homeostasis through the regulation of hepatic uptake of triglycerides and glucose and stimulation of lipolysis in the adipose tissue^32^. GH deficiency is known to recapitulate the aberrant metabolic profile of MetS (i.e., hypertriglyceridemia, low HDL-C, insulin resistance)^33–35^. We have demonstrated that presentation of CO or the other 4-risk factor cluster alone reduced the level of circulating GH. Intriguingly, this reduction was only exacerbated by the other 4 risk factors in CO individuals but not the *vice versa*. Considering that AG is a known potent secretagogue of GH^36^, the reduction of GH associated with the other 4 risk factors could be an effect secondary to the decrease in AG. However, this notion is not supported by our data that CO decreased the level of GH without affecting the circulating AG; implying that the down-regulation of GH by CO is probably mediated in a ghrelin-independent fashion. For instance, it has been reported previously that adiponectin regulates GH secretion through adiponectin receptor followed by Ca^2+^ signalling^37^. Consistent findings from our laboratory have also revealed an association between CO and reduced circulating adiponectin^38^.

Increasing prevalence of CO and MetS arouses the need to comprehend the resultant physiological significance. According to our data, CO in conjunction with other MetS risk factors did not modulate necessarily the same signalling peptides (i.e., AG, UnAG, obestatin, GH and obestatin/ghrelin ratio) in the ghrelin signalling and GH/IGF-1 axis. While both CO and the cluster of the four MetS risk factors are associated with aberrant alterations of GH and obestatin, the changes in serum levels of UnAG, AG and total ghrelin are only mediated by the cluster of four MetS risk factors. Although CO is considered as an essential risk factor of MetS according to the definition of IDF, our data do not support to use CO solely in the prediction of dysregulation of MetS-related signalling (e.g., ghrelin signalling). The pathogenic outcome is likely to be determined by intricate orchestration between CO and other MetS factors. Future research should aim to decipher the contributions of respective and different combinations of MetS risk factors and the underlying signalling pathways in the progression of MetS and subsequent development of chronic diseases such as cardiovascular diseases and type 2 diabetes mellitus.

## Supplementary information

Supplementary Data.
