# Supplementary material for: Obestatin and growth hormone reveal the interaction of central obesity and other cardiometabolic risk factors of metabolic syndrome
Source: Sci Rep. 2020 Mar 26;10:5495. doi: 10.1038/s41598-020-62271-w (PMC7099091; doi:10.1038/s41598-020-62271-w)
Supplement: Supplementary file 1 — Supplementary Data. [file 41598_2020_62271_MOESM1_ESM.docx]

**Obestatin and growth hormone reveal the interaction of central obesity and other cardiometabolic risk factors of metabolic syndrome**

Angus P. Yu^1^, Felix N. Ugwu^3^, Bjorn T. Tam^2^, Paul H. Lee^4^, Vicki Ma^3^, Simon Pang^3^, Angel S. Chow^3^, Kenneth K. Cheng^3^, Christopher W. Lai^5^, Cesar S. Wong^3^, and Parco M. Siu^1^*

^1^Division of Kinesiology, School of Public Health, Li Ka Shing Faculty of Medicine, The University of Hong Kong, Pokfulam, Hong Kong, China

^2^Department of Health, Kinesiology and Applied Physiology, Concordia University, Montreal QC, Canada

^3^Department of Health Technology and Informatics, Faculty of Health and Social Sciences, The Hong Kong Polytechnic University, Hung Hom, Kowloon, Hong Kong, China

^4^School of Nursing, Faculty of Health and Social Sciences, The Hong Kong Polytechnic University, Hung Hom, Kowloon, Hong Kong, China

^5^Singapore Institute of Technology, Singapore

**SUPPLEMENTARY DATA**

**Supplementary data**

Immunoblotting for GH and obestatin was conducted to validate the results of ELISA. Sera were boiled by 95°C with 5% beta-mercaptoethanol for 15 minutes. Equal volume of sera were load on 15% polyacrylamide gel. Proteins were transferred to polyvinylidene difluoride membranes (80 volts for 120 minutes) after electrophoresis. The membranes were then blocked with 5% non-fat milk for 1 hour at room temperature followed by overnight incubation at 4°C with corresponding primary antibody (anti-growth hormone polyclonal antibody, 1:1000 dilution, ab109759, Abcam; anti-obestatin monoclonal antibody, 1:1000 dilution, ab216575, Abcam). Membranes were then washed in TBST for three times and incubated with horseradish peroxidase (HRP)-conjugated secondary antibody at room temperature for 1 hour (anti-rabbit IgG antibody, 1: 4000 dilution, 7074, Cell Signaling Technology). Luminol reagent (NEL103001EA; Perkin Elmer, Waltham, MA, USA) was then applied to the membranes for chemiluminescence detection of HRP. Chemiluminescence signal was detected and captured using ImageQuant LAS 4000mini, GE healthcare). The resulting bands were quantified by ImageJ as optical density (OD) X band area.

**Supplementary figure 1 Validation of ELISA results of obestatin and growth hormone using immunoblotting**

A


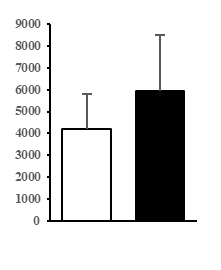


**-**

**(n=86)**

**+**

**(n=47)**


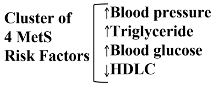


**p < 0.001**


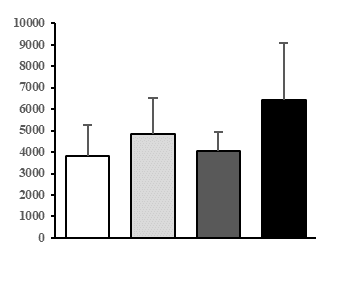


C


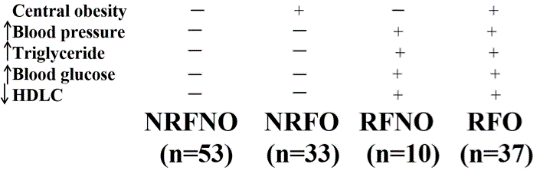


**Obestatin (Arbitrary unit)**


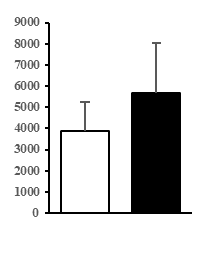


B

**-**

**(n=63)**

**+**

**(n=70)**

**Central obesity**

**Obestatin (Arbitrary unit)**

**p < 0.001**

**p < 0.001**

**p = 0.030**

**Obestatin (Arbitrary unit)**

**p < 0.001**

**p < 0.001**

**
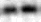

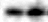
**

**Obestatin (13kDa)**

**Obestatin (13kDa)**

**
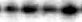
**

**Obestatin (13kDa)**

D

**-**

**(n=86)**

**+**

**(n=47)**


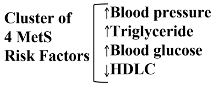


**Growth hormone**

**(Arbitrary unit)**

E

**-**

**(n=63)**

**+**

**(n=70)**

**Central obesity**

**Growth hormone**

**(Arbitrary unit)**

F

**p < 0.001**

**p < 0.001**

**p < 0.001**

**p < 0.001**

**p < 0.025**

**Growth hormone**

**(Arbitrary unit)**


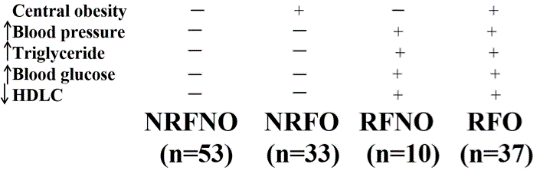


**p = 0.049**


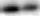

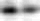


**Growth hormone (22kDa)**

**Growth hormone (22kDa)**

**
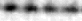
**

**Growth hormone (22kDa)**

**Supplementary figure 1 Validation of ELISA results of obestatin and growth hormone using immunoblotting**

Obestatin level in subjects with/without the cluster of four MetS risk factors regardless of the status of central obesity (RFNO and RFO vs NRFNO and NRFO) (A). Obestatin level in subjects with/without central obesity regardless of the status of the cluster of four MetS risk factors (NRFNO and RFNO vs NRFO and RFO) (B). Comparison of obestatin among subjects with no MetS risk factors, central obese only, the cluster of the other MetS risk factors other than central obese and all MetS risk factors. (C). Growth hormone level in subjects with/without the cluster of four MetS risk factors regardless of the status of central obesity (RFNO and RFO vs NRFNO and NRFO) (D). Growth hormone level in subjects with/without central obesity regardless of the status of the cluster of four MetS risk factors (NRFNO and RFNO vs NRFO and RFO) (E). Comparison of growth hormone level among subjects with no MetS risk factors, central obese only, the cluster of the other MetS risk factors other than central obese and all MetS risk factors (F)
